# Impact of Normothermic Preservation with Extracellular Type Solution Containing Trehalose on Rat Kidney Grafting from a Cardiac Death Donor

**DOI:** 10.1371/journal.pone.0033157

**Published:** 2012-03-21

**Authors:** Satomi Iwai, Takeshi Kikuchi, Naoya Kasahara, Takumi Teratani, Takashi Yokoo, Iwao Sakonju, Shouzou Okano, Eiji Kobayashi

**Affiliations:** 1 Laboratory of Small Animal Surgery I, School of Veterinary Medicine, Kitasato University, Aomori, Japan; 2 Center for Development of Advanced Medical Technology, Jichi Medical University, Tochigi, Japan; 3 Project Laboratory for Kidney Regeneration, Institute of DNA Medicine, Department Internal Medicine, The Jikei University School of Medicine, Tokyo, Japan; 4 Laboratory of Small Animal Surgery II, School of Veterinary Medicine, Kitasato University, Aomori, Japan; University of Colorado, United States of America

## Abstract

**Background:**

The aim of this study was to investigate factors that may improve the condition of a marginal kidney preserved with a normothermic solution following cardiac death (CD) in a model of rat kidney transplantation (RTx).

**Methods:**

Post-euthanasia, Lewis (LEW) donor rats were left for 1 h in a 23°C room. These critical kidney grafts were preserved in University of Wisconsin (UW), lactate Ringer's (LR), or extracellular-trehalose-Kyoto (ETK) solution, followed by intracellular-trehalose-Kyoto (ITK) solution at 4, 23, or 37°C for another 1 h, and finally transplanted into bilaterally nephrectomized LEW recipient rats (n = 4–6). Grafts of rats surviving to day 14 after RTx were evaluated by histopathological examination. The energy activity of these marginal rat kidneys was measured by high-performance liquid chromatography (HPLC; n = 4 per group) and fluorescence intensity assay (n = 6 per group) after preservation with UW or ETK solutions at each temperature. Finally, the transplanted kidney was assessed by an *in vivo* luciferase imaging system (n = 2).

**Results:**

Using the 1-h normothermic preservation of post-CD kidneys, five out of six recipients in the ETK group survived until 14 days, in contrast to zero out of six in the UW group (*p*<0.01). Preservation with ITK rather than ETK at 23°C tended to have an inferior effect on recipient survival (*p* = 0.12). Energy activities of the fresh donor kidneys decreased in a temperature-dependent manner, while those of post-CD kidneys remained at the lower level. ETK was superior to UW in protecting against edema of the post-CD kidneys at the higher temperature. Luminescence intensity of successful grafts recovered within 1 h, while the intensity of grafts of deceased recipients did not change at 1 h post-reperfusion.

**Conclusions:**

Normothermic storage with extracellular-type solution containing trehalose might prevent reperfusion injury due to temperature-dependent tissue edema.

## Introduction

Renal transplantation (RTx) offers prolonged survival and improved quality of life for patients with end-stage renal disease who are facing dialysis [1]. To bridge the growing gap between organ supply and demand, recent efforts have relied on donors considered to be less than optimal, including those who are older, have experienced cardiac death (CD), and those with comorbidities that are potentially detrimental to long-term graft survival [2]–[5]. Despite the increasing use of organs from such marginal donors, waiting lists and deaths while awaiting RTx have been on the rise [6]. Overall, marginal RTx are limited by inferior outcomes and higher costs [6].

Compared with organs from living and brain-dead donors, kidneys from CD donors (CDD) are significantly more prone to ischemia/reperfusion injury, which may account for the higher rates of both primary non-function and delayed graft function (DGF) [3], [7]. Indeed, the presence of DGF increases the risk of an acute rejection and doubles the rate of graft loss within 5 years [8], [9].

To maintain the function of marginal organs, various preservation methods have been developed and promoted [10]–[13]. Although the use of hypothermic conditions in order to reduce metabolism and oxygen demand has been a fundamental principle in clinical organ preservation [10], several normothermic preservation and perfusion methods have recently been proposed [10], [14]–[16]. For instance, a warm flush can be used to achieve more rapid clearance of blood from the microcirculation, whereas a warm preservation solution may reduce vasoconstriction and prevent cell membrane stiffening of both endothelial cells and the cellular components of the blood [14]. These actions may then ameliorate the no-reflow phenomenon, an important mechanism in ischemia/reperfusion injury [14]. Unfortunately, rewarming induces ultrastructural changes in renal tubular cells similar to those observed in acute tubular necrosis, which are associated with renal failure [17]. Thus, further investigations of the effects of normothermic preservation of grafts from marginal donors are clearly warranted.

ET-Kyoto Solution (ETK) was originally developed for use in preserving lung tissue [18], [19]. ETK contains trehalose and gluconate, two distinct components which have a cytoprotective effect and prevent cells from swelling [20], [21]. Since ETK has a high-sodium/low-potassium composition akin to extracellular fluid, it prevents the vasoconstriction induced by high potassium concentration and cellular edema [22], [23]. In addition, ETK has other advantages, including the ability to be stored at room temperature for 3 years [22]. This characteristic of ETK could provide significant cost savings compared with UW.

Genetically encoded biological light probes can be used as high-performance tools to visualize spatiotemporal tissue fate in living animals. *In vivo* luciferase imaging is obtained from luciferase-expressing LEW transgenic rats via the use of a noninvasive bioimaging system [23]. Due to a close correlation between the two factors, a change in ATP activity can be quantified according to fluorescence intensity [24].

The purpose of this study was to investigate whether a post-CD kidney could be transplanted following prolonged control of preservation temperatures using ETK in a rat RTx model. The effect of normothermic preservation with ETK was compared to those of preservation with University of Wisconsin (UW) and intracellular-trehalose-Kyoto (ITK) solution.

## Results

### Survivals after RTx from CD donors

Survival rates of all rats post-RTx from CD donors are summarized in Table 1. In experiment 1, all rats implanted with grafts that were left for 1 h following CD (CD1h group) survived. However, all rats implanted with grafts that were left for 2 h after CD (CD2h group) died within 5 days. Thus, the survival rate of the CD1h group was significantly greater than that of CD2h group (*p*<0.01). Next, we investigated various methods that could extend the preservation period of CD1h.

**Table 1:** Differentiation of survival time under each preservation condition.

		Preservation condition				
	Time after cardiac death(h)	Time(h)	Temperature(°C)	Preservation solution	N	Post-transplantation survival(day)
**Experiment1**	1	0	-	-	5	>14, >14, >14, >14, >14
	2	0	-	-	5	2, 3, 3, 4, 5*
**Experiment2**	1	1	4	ETK	6	3, 3, 3, 4, 4, 4#
	1	1	22–23	ETK	6	5, >14, >14, >14, >14, >14
	1	1	37	ETK	4	2, 2, 3, 3#
	1	1	4	LR	4	3, 3, 3, 4#
	1	1	22–23	LR	6	2, 3, 3, 3, 4, >14#
	1	2	22–23	ETK	4	2, 3, 3, 5#
**Experiment3**	1	1	4	UW	6	2, 2, 2, 4, 4, >14##
	1	1	22–23	UW	6	2, 2, 3, 3, 3, 5# †
	1	1	22–23	ITK	6	5, 6, 6, 12, >14, >14

* vs. CD1h *p*<0.01 using Log-Rank test.

# vs. ETK23 *p*<0.01 using Log-Rank test.

## vs. ETK23 *p*<0.05 using Log-Rank test.

† vs. ITK23 *p*<0.01 using Log-Rank test.

ETK, ET-Kyoto solution; UW, University of Wisconsin solution; LR, lactate Ringer's solution; ITK, IT-Kyoto solution.

In experiment 2, the survival rate of the ETK-preservation group at 23°C (ETK23 group; 12.2±4.0 days) was significantly greater than that of all the other groups (ETK at 4°C [3.7±0.6 days], ETK at 37°C [2.7±0.6 days], LR at 4°C [3.0±1.0 days], LR at 23°C [6.3±5.2 days], and the 2-h ETK preservation at 23°C [3.3±1.3 days]; *p*<0.01 for all comparisons). Our results suggest normothermic preservation as an optimal method for extending the viability of CD1h.

In experiment 3, we assessed the effect of the preservation temperatures using UW and ITK. Only one recipient survived in response to preservation with UW at 4°C (4.7±4.7 days). However, no recipient survived beyond 5 days post-RTx under normothermic preservation with UW (average, 3±1.1 days). Preservation with ITK at 23°C (ITK23) resulted in a higher survival rate compared to UW at 4°C (*p* = 0.09) and UW at 23°C (*p*<0.01). Although ITK23 preservation (9.5±4.3 days) also tended to have an inferior effect on the recipient's survival in comparison to ETK, no significant differences were observed.

### Blood assay after RTx

**Figure 1:**
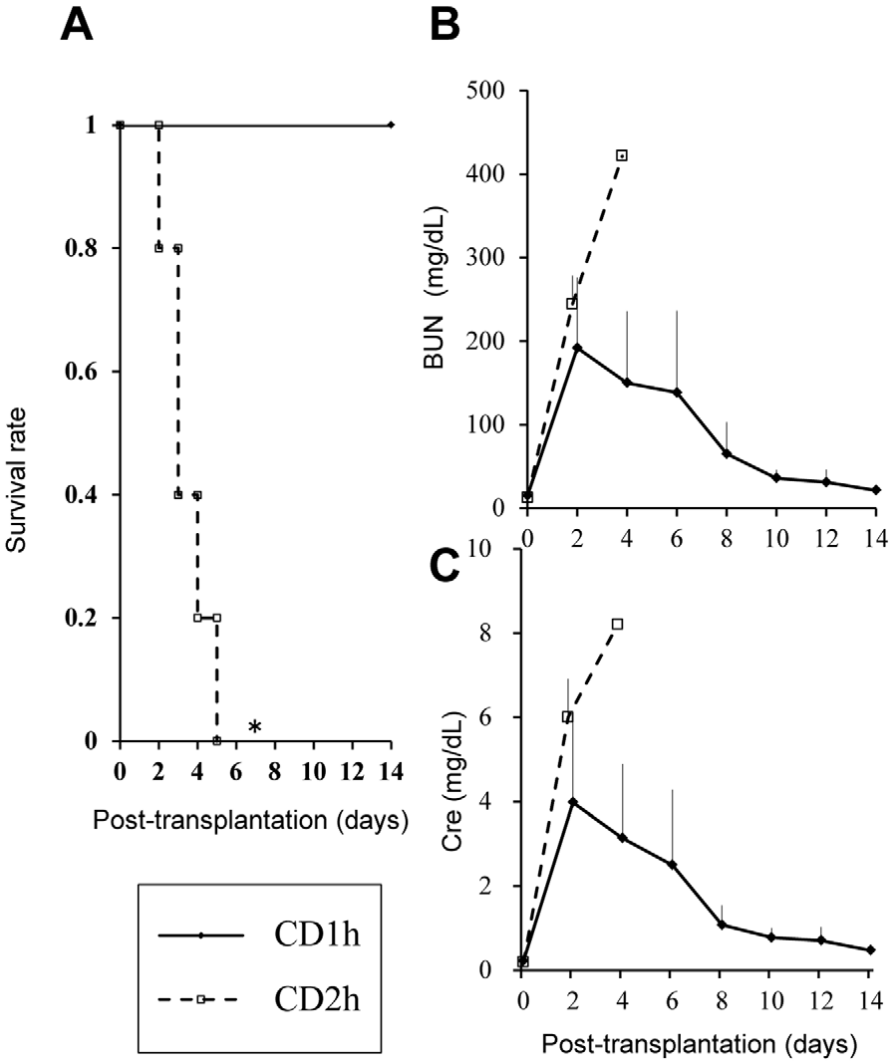
Survival rate, blood urea nitrogen (BUN) and serum creatinine (Cre) levels in experiment 1. (A): Survival rate of each group. Survival rate of the CD1h group was significantly greater than that of the 2-h post-CD group (* *p*<0.01 using Log-Rank test.). (B): BUN. (C): Cre. BUN and Cre levels of all surviving rats were gradually recovered to a normal range by day 14 after RTx.

**Figure 2:**
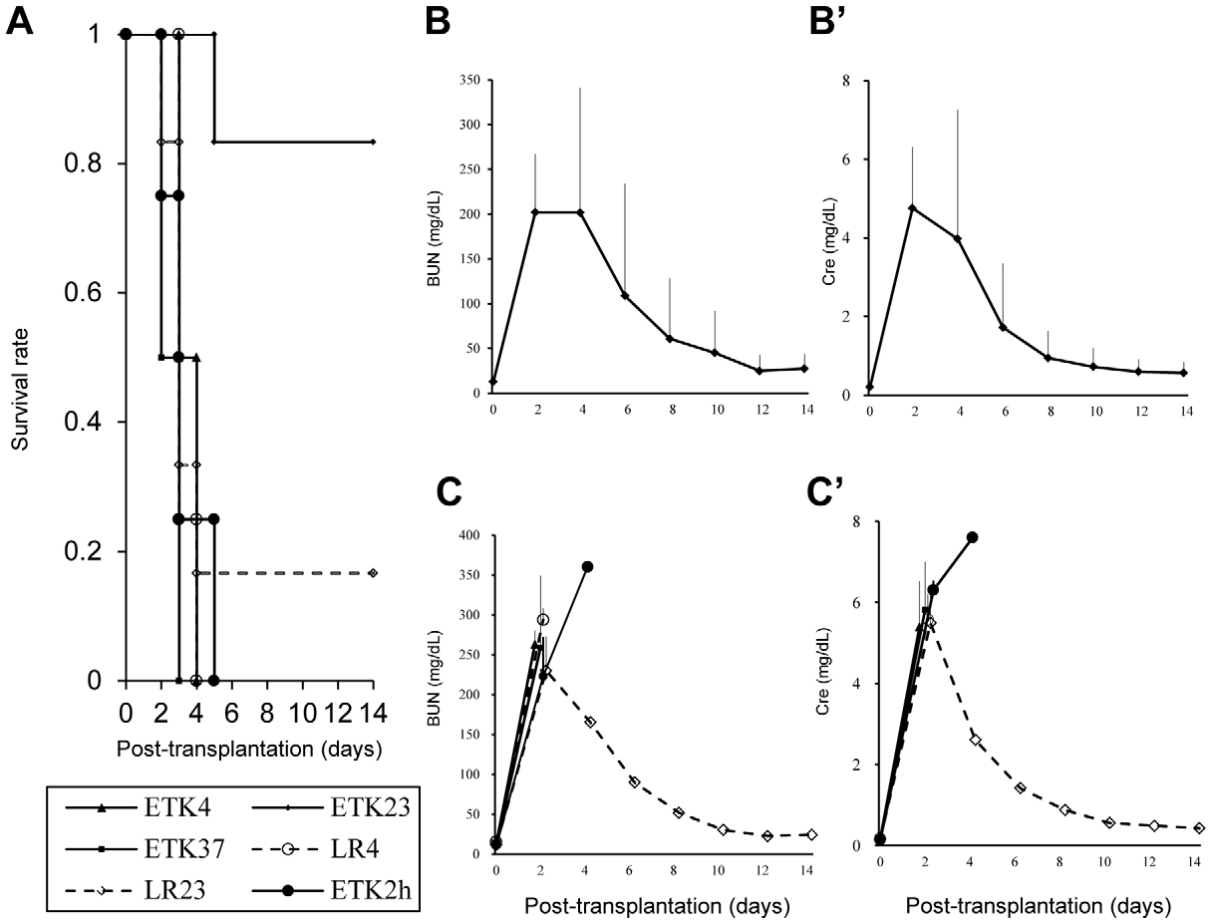
Survival rate, blood urea nitrogen (BUN) and serum creatinine (Cre) levels in experiment 2. (A): Survival rate of each group. Survival rate of the group receiving kidneys preserved with ET-Kyoto solution (ETK) for 1 h at 23°C (ETK23 group) was significantly longer than that of the other groups (# *p*<0.01 using Log-Rank test.). (B): BUN and (B'): Cre of the ETK23 group. (C): BUN and (C'): Cre of the other groups without EK23. In all groups in experiment 2, BUN and Cre of all surviving rats reached a peak at day 2 and recovered to a nearly normal range by day 14. (BUN and Cre of the LR23 group was n = 1 after day 4.)

**Figure 3:**
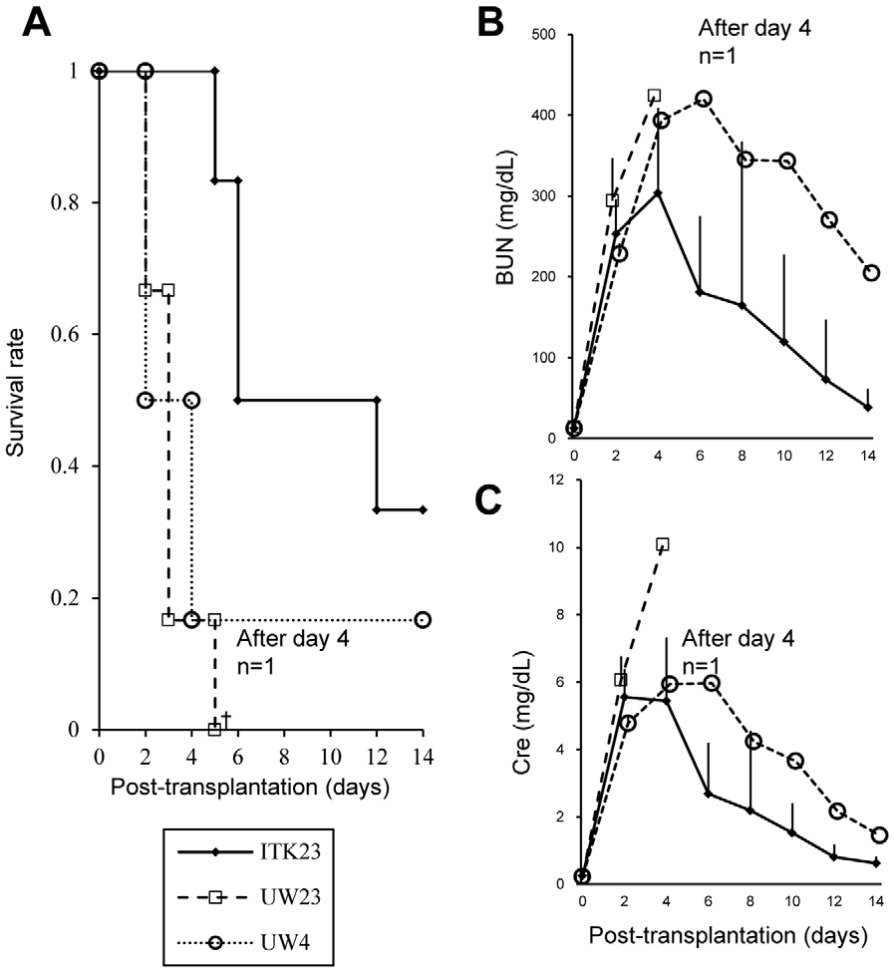
Survival rate, blood urea nitrogen (BUN) and serum creatinine (Cre) levels in experiment 3. (A): Preservation with ITK at 23°C (ITK23) tended to increase the survival rate compared to UW at 4°C (*p* = 0.09) and UW at 23°C (*p*<0.01). (B): BUN and Cre levels of all surviving rats were gradually recovered to a normal range by day 14 after RTx. (BUN and Cre levels of the UW4 group were based on a single animal after day 4.)

Changes in blood urea nitrogen (BUN) and serum creatinine (Cre) post-RTx for experiments 1, 2, and 3 are shown in Figures 1, 2, and 3, respectively. Regardless of experimental group, levels of BUN and Cre of all surviving rats reached a peak at day 2 and recovered to a nearly normal range by day 14. However, levels of both BUN and Cre of deceased rats tended to increase with elapsed time.

### Pathological status of kidney graft after RTx

**Figure 4:**
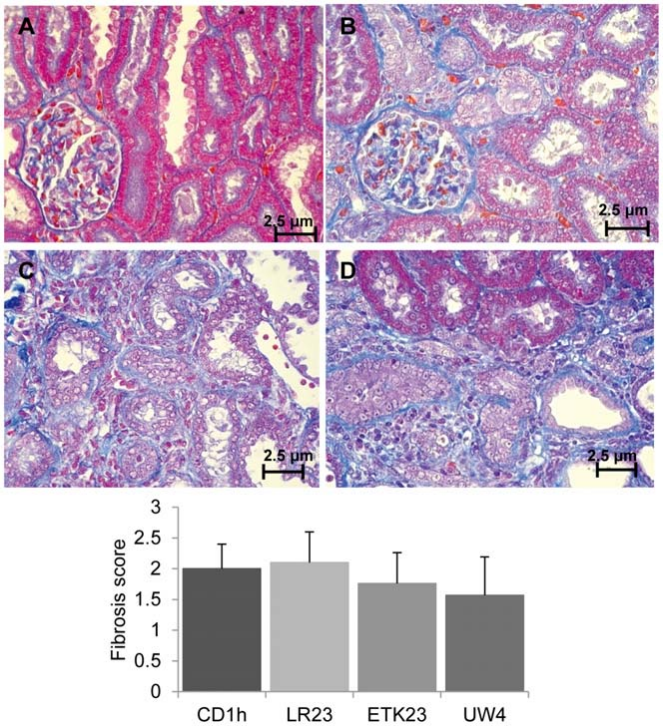
Pathological status of kidney graft after kidney transplantation (RTx). After euthanasia on day 14 after RTx, kidneys were harvested and stained with azan stain for parenchymal fibrosis. Fibrosis was graded numerically as follows; (A): 0 = none, (B): 1 = minimal, (C): 2 = moderate, and (D): 3 = severe. In each slide, 20 fields were evaluated by four observers blinded to treatment (4×20 fields). Fibrosis of the group receiving kidneys preserved with ETK23 tended to be lower than that of the other groups, but the difference was not significant (*p*>0.05).

Only kidneys of rats surviving until day 14 were confirmed using azan stain to investigate fibrotic level. Even the graft kidneys of the CD1h group were replaced by fibrosis, suggesting severe tissue injury. The pathological scores of the CD1h alone, ETK23, UW4, and CD1h followed by LR preservation at 23°C for 1 h (LR23) groups were 2.0±0.4, 1.76±0.5, 1.57±0.6, and 2.1±0.5, respectively. The tissue fibrosis of the ETK23 and UW4 groups tended to be inhibited in contrast to that of the other groups, although these differences were not statistically significant (Figure 4). Thus, it remains possible that ETK and UW reduce tissue injury of the marginal kidney.

### Gravimetric measurements of kidney weights

**Figure 5:**
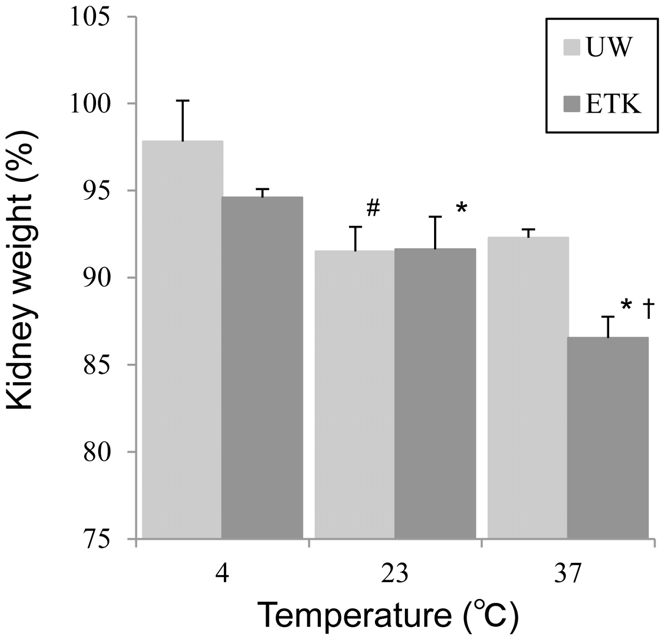
Gravimetric measurements of kidney weights. ETK significantly reduced the kidney weights (KW) with increasing preservation temperature (* ETK at 23°C vs. ETK at 4°C, *p*<0.05; and ETK at 23°C vs. ETK at 37°C, *p*<0.001). The prevention of cellular edema with the UW solution was less pronounced as compared to ETK (# UW at 23°C vs. UW at 4°C, *p*<0.01; and UW at 23°C vs. UW at 37°C, *p*<0.05). At 37°C, the KW of ETK was significantly lower than that of UW († *p*<0.001).

CD kidney weight was measured to evaluate tissue edema in response to use of ETK and UW at 4, 23 and 37°C (Figure 5). In contrast to ETK preservation at 4°C, the donor kidney weight was significantly lower with ETK at 23°C (*p*<0.05) and ETK at 37°C (*p*<0.001). Although tissue edema was also significantly reduced with UW at 23°C (*p*<0.01) and UW at 37°C (*p*<0.05) in contrast to UW at 4°C, this effect tended to be less pronounced than with ETK. For instance, at 37°C, the kidney weights of ETK were significantly lower than that of UW (*p*<0.001).

### Analysis of energy charge in preserved rat kidney

**Figure 6:**
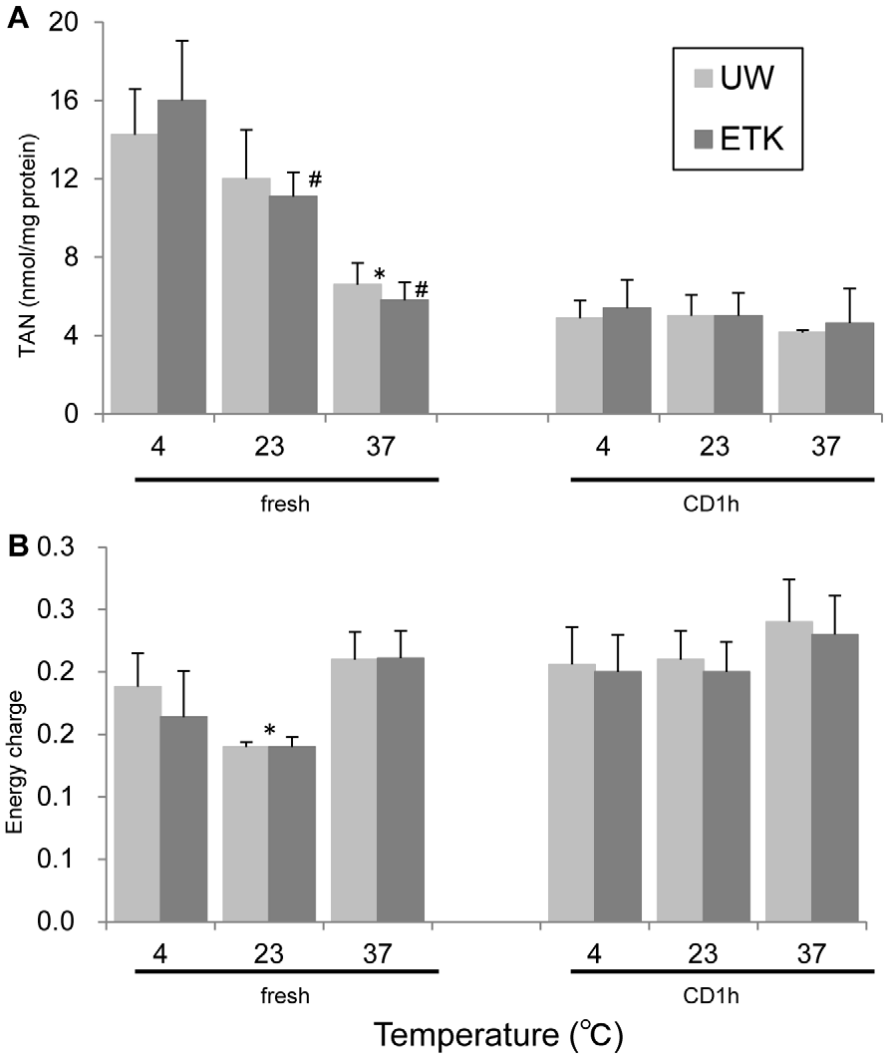
Impact of each preservation solution and temperature on tissue TAN and EC of preserved rat kidney. (A): The TAN of fresh kidneys at 37°C and CD1h at all temperatures were lower than that of fresh kidneys at 4°C and 23°C (* *p*<0.05, fresh37 vs. fresh4 in UW; # *p*<0.05, fresh37 vs. fresh4 and 23 in ETK). (B) The EC of the fresh37, CD1h4, CD1h23, and CD1h37 groups was not higher than that of the fresh4 and fresh23 groups. The EC of fresh23 with UW preservation was significantly lower than that of fresh4 (* *p*<0.05).

Total adenine nucleotide level (TAN, nmol/mg protein) and energy charge (EC) of kidneys are shown in Figure 6. TAN levels of fresh kidneys at 37°C (fresh37) and CD1h kidneys at all temperatures tended to be lower than that of fresh kidneys at 4°C and 23°C (fresh4 and fresh23, respectively). Specifically, the TAN level of fresh37 kidneys was significantly lower than that of fresh4 kidneys in UW-preservation groups (*p*<0.05). Additionally, the TAN levels of fresh23 and fresh37 kidneys were significantly lower than that of fresh4 kidneys in ETK-preservation groups (*p*<0.05 and *p*<0.01, respectively).

Using ETK preservation, the EC of fresh37, CD1h4, CD1h23, and CD1h37 kidneys tended to be higher than that of fresh4 and fresh23 kidneys. With UW preservation, the EC of fresh23 kidneys was significantly lower than that of fresh4 kidneys (*p*<0.05).

### Determination of fluorescence intensity of kidney chips

**Figure 7:**
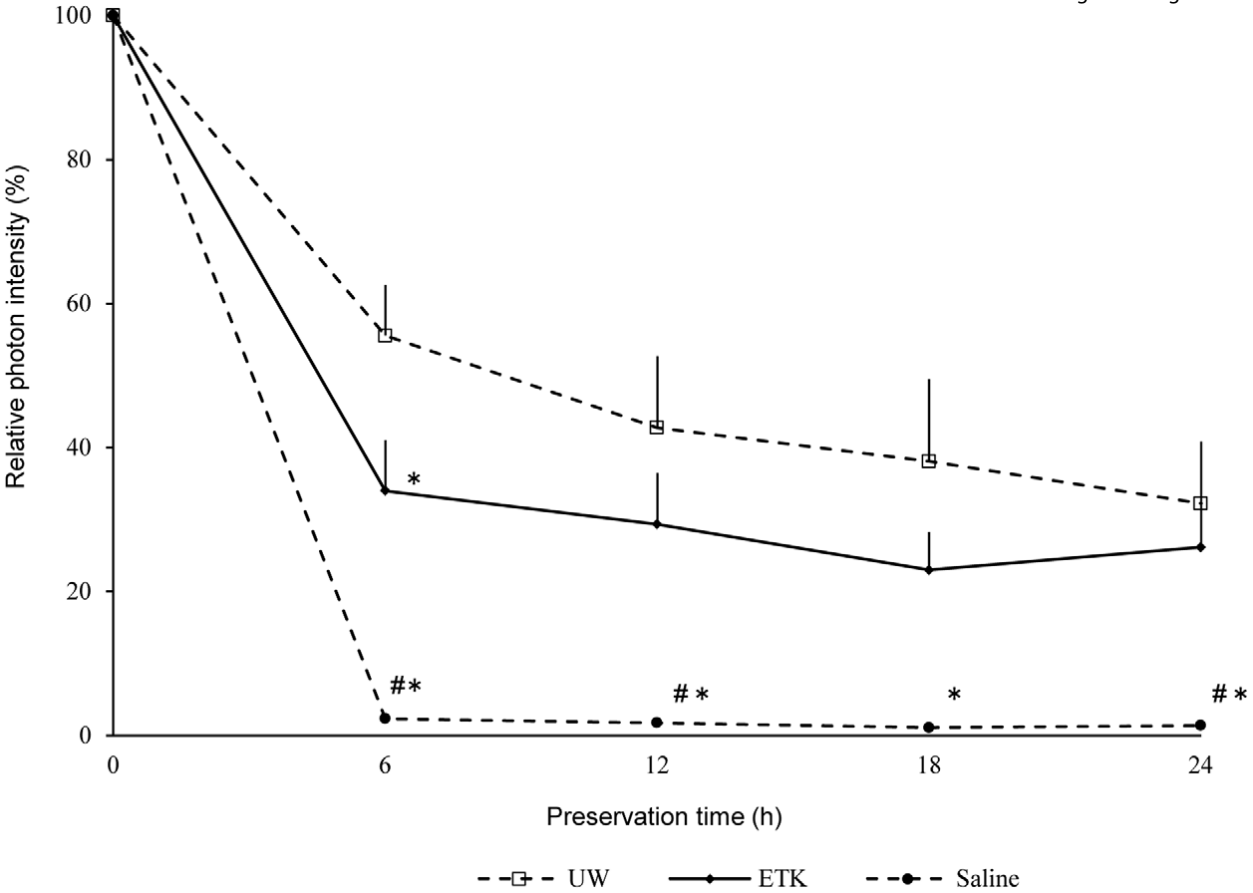
Determination of fluorescence intensity of kidney chips. The fluorescence intensity of the UW group at 6 h after preservation was significantly higher than that of the ETK group (* *p*<0.05). However, the fluorescence intensity of both groups was approximately equal at 24 h. (# *p*<0.05 vs. ETK group).

Changes in the fluorescence intensity of kidney chips preserved at 4°C are shown in Figure 7. The fluorescence intensity of the UW group at 6 h after preservation (55.54±17.21%) was significantly higher than that of the ETK group (34.00±37.33%, *p*<0.05). However, the fluorescence intensity of both groups was approximately equal at 24 h (ETK: 26.17±18.63%, UW: 32.23±21.04%, *p*>0.05). Finally, fluorescence intensities of ETK and UW were significantly higher than that of saline at almost all measurement time points (*p*<0.05).

### Changes in luminescence intensity of graft kidney after RTx using Luc-Tg rat

**Figure 8:**
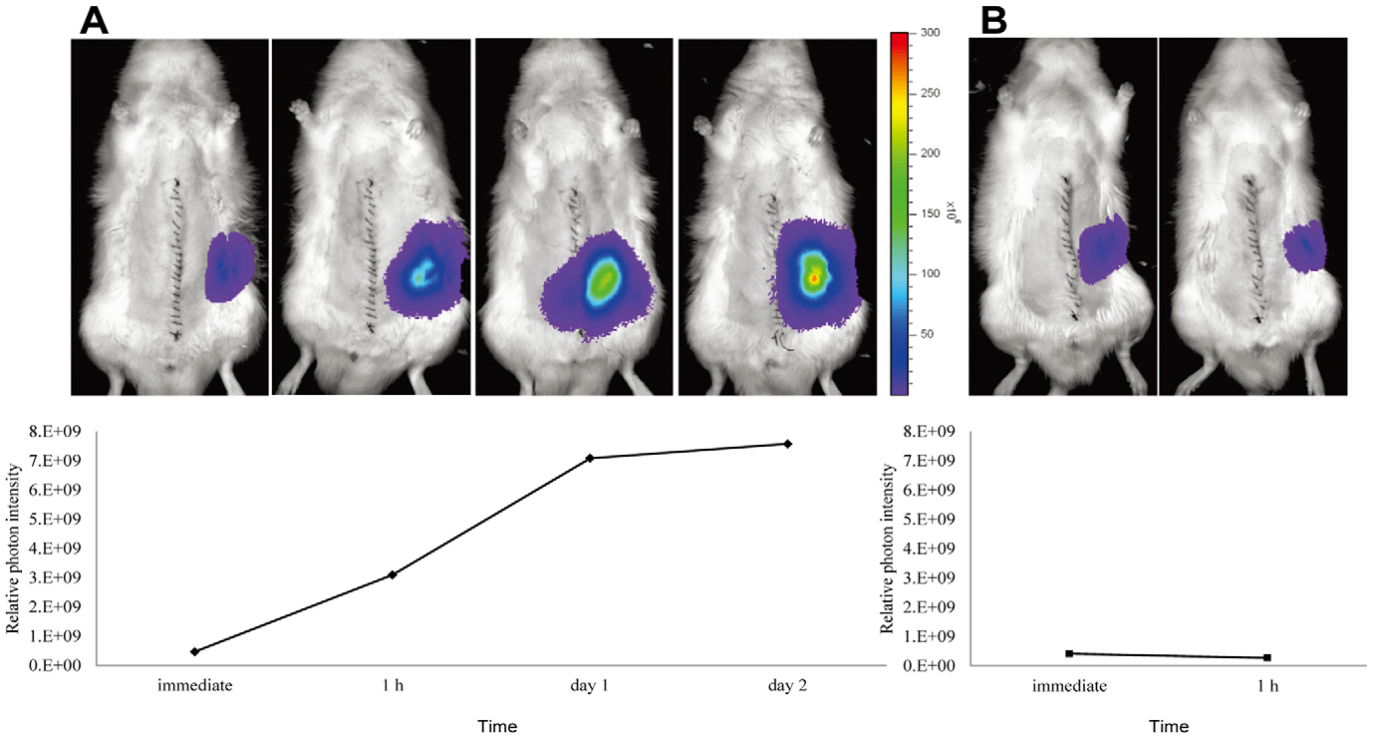
Comparison of change in luminescence intensity between fresh and critical kidney grafts. (A): Photon flux of rat transplanted fresh kidney began to increase at 1 h after RTx, and stabilized after 1 day. (B): Photon flux of the critical kidney was unchanged and the recipient rat did not survive.

Similar to that observed for the DC1h donor kidney, the photon flux of rat transplanted fresh kidney began to increase from a low level at 1 h after RTx, and subsequently stabilized after 1 day (Figure 8-A). However, the photon flux of the critical kidney was unchanged and the recipient rat did not survive (Figure 8-B).

## Discussion

In the present study, we investigated whether a post-CD donor kidney can function after various prolonged preservation conditions, including variations in the temperature of the preservation solution. Secondly, we studied whether the energy activity of kidneys after CD or graft kidney after reperfusion during RTx was influenced by the type of preservation solution.

We observed that the survival rate of the ETK group was superior to that of the UW and ITK groups at the normothermic temperature; a finding possibly explained by a difference in sodium/potassium composition in the solutions. Specifically, while ETK has a high-sodium/low-potassium composition similar to that of extracellular fluid, UW has a low-sodium/high-potassium composition similar to that of intracellular fluid (Table 2) [25]. Furthermore, Faure et al. reported that compared to cold preservation using traditional UW solution, a high-sodium version of UW decreased GFR, urinary protein, interstitial fibrosis, and CD4+-cell infiltration in a pig auto-RTx model [26]. These findings support our result of a superior survival rate among ETK versus ITK groups. Therefore, extracellular fluid was better adapted to preserve the marginal kidney compared with intracellular fluid at the normothermic state.

**Table 2:** Compositions of ETK, ITK, UW or LR.

	ETK	ITK	UW	LR
**Na (mmol/L)**	**100**	**20**	**30**	130
**K (mmol/L)**	**44**	**130**	**125**	4
**Cl (mmol/L)**	-	-	-	109
**Mg (mmol/L)**	-	-	5	-
**Ca (mmol/L)**	-	-	-	1.5
**Gluconate (mmol/L)**	**100**	106	-	-
**Phosphate (mmol/L)**	25	25	25	-
**Sulfate (mmol/L)**	-	-	5	-
**Lactobionate (mmol/L)**	-	-	100	-
**Lactate (mmol/L)**	-	-	-	28
**Raffinose (mmol/L)**	-	-	**30**	-
**Adenosine (mmol/L)**	-	-	5	-
**Alloprinol (mmol/L)**	-	-	1	-
**Trehalose (mg/L)**	**41**	**41**	-	-
**Hydroxyethyl starch (mg/L)**	30	30	50	-
**Glutathion (mmol/L)**	-	-	3	-
**Osmorality (mOsm/L)**	366	370	320	250

Another key consideration of normothermic preservation is the prevention of cellular edema of the critical organ. According to available evidence, tissue fibrosis appears to be associated with cellular edema [23], [26]. Interestingly, carbohydrates, such as the trehalose found in ETK and the raffinose found in UW, might inhibit the development of cellular edema. The difference in kidney weight following preservation also has an impact on the cellular edema produced from ischemia/reperfusion injury [27]. In our kidney weight measurement assay, graft weight was increased by preservation with ETK in a temperature-dependent matter. However, UW was less effective in this regard. Trehalose provides a larger monolayer lateral expansion and is substantially superior to raffinose in terms of the stabilization of proteins and nucleic acids [20], [28]–[30]. Furthermore, the phase transition temperature is 24°C with ETK, but 17°C with raffinose [31], [32]. Based on evidence from canine and human reports, normothermic perfusion provided life-sustaining function, whereas hypothermic machine perfusion failed [16], [33]. In the current study, we observed that normothermic temperatures were optimal for preservation with ETK. Additionally, at these temperatures, the ETK and ITK preservation groups exhibited a significantly higher survival rate than the UW preservation group. Thus, it was revealed that normothermic preservation with a trehalose-containing solution was important for maintaining the health of a marginal kidney.

Using an *in vitro* assay, we showed significantly decreased TAN levels of the CD1h versus fresh kidney. During hypoxia, AMP accumulation and EC is stabilized by a reduction in the TAN pool through a degradation of catalyzed AMP [34], [35]. While our results were in general consensus with these observations, we found no difference in EC between ETK and UW solution preservation at different temperatures. The EC at which vertebrates can survive is 0.5–0.6 [35]. Thus, since the EC of all groups was similar, we suspected that the EC during preservation is not solely associated with survival after reperfusion of the marginal kidney.

Using the marginal Luc-Tg rat RTx system, we also confirmed the importance of immediately increasing ATP following reperfusion for the survival of recipient rats. This suggests that the key to improving the survival of the marginal kidney is a rapid recovery of EC after reperfusion. The energy state is affected by warm ischemia and subsequent recirculation [36]. Adequate cellular ATP is required for reestablishing ionic homeostasis and supporting ATP-dependent membrane pump function during the critical reperfusion period [37]. Cold preservation results in poor oxygen supply and delayed reduction in the levels of vasoconstrictive arachidonate substrate; actions that result in weak vasodilatation and a delayed recover of EC after reperfusion following cold storage [38], [39]. While cold temperature causes necrosis, the consequent re-warming can induce apoptosis, which leads to the further development of tissue fibrosis [39]. Salahudeen et al. reported that prolonged cold ischemia of cadaveric grafts is a significant predictor of long-term graft loss [40]. The Na^+^/K^+^ ATPase of the kidney is particularly sensitive to ATP, where the activity of proximal tubules is in direct proportion to ATP concentration [41]. Therefore, hypothermia can lead to severe injury of the proximal tubules. Thus, we surmise that the biggest factors influencing survival of the marginal kidney are an early recovery of EC and the prevention of cellular edema using normothermic preservation. Furthermore, we observed that the passive prevention of edema using carbohydrates (trehalose) was more effective than active prevention of edema for purposes of preserving a marginal kidney with energy depletion.

In conclusion, normothermic preservation with ETK, an extracellular-type solution containing trehalose, was beneficial for the short-term storage of a marginal kidney. This approach will be able to extend the preservation period of marginal kidneys or to provide improved function of marginal kidneys for longer periods.

## Materials and Methods

### Animals

Male wild LEW rats weighing between 260 and 310 g werepurchased from Charles River (Breeding Laboratories, Kanagawa, Japan). The animals were housed in a temperature- and humidity-controlled environment with a 12-h light/dark cycle and were provided with standard laboratory chow and water *ad libitum*. Luc-Tg LEW rats, produced from cross-breeding normal LEW rats (Charles River Laboratories Japan, Inc., Kanagawa, Japan) and Luc-Tg rats, were used for the experiments [42]. Rats were fed and bred at the Center for Experimental Medicine, Jichi Medical University. All experiments were conducted by following established guidelines for animal welfare and were approved by the animal ethics committee of Kitasato University (09-149) and Jichi Medical University (1111).

### Transplantation procedure

Post-euthanasia, donor rats were left in the 23°C room for a specified time before the left kidney was harvested. The graft kidneys were transplanted into the recipients according to conditions of preservation solutions and timing as described in Table 1.

In experiment 1, LEW rats were sacrificed, and their left kidneys were harvested after 1 or 2 h following CD. Kidneys that were not preserved with any solutions were immediately transplanted into bilaterally nephrectomized recipients.

In experiments 2 and 3, ETK, ITK, LR (Otsuka Pharmaceutical Factory Inc., Naruto, Japan), and UW (ViaSpan, DuPont Pharmaceuticals, Wilmington, DE, USA), were used as the preservation solutions. Components of each solution are shown in Table 2. For each preservation solution, the grafts of recipients were either placed into refrigerators for storage, left in a room maintained at 23°C, or placed in a constant-temperature bath controlled at 37°C. During the preservation period, anesthesia of recipient rats was induced using isoflurane and was maintained throughout surgery. The donor renal artery was anastomosed to the aorta of the recipient, while the renal vein was connected to the inferior vena cava using a side-to-end anastomosis with a continuous running suture. The ureter was anastomosed end-to-end using interrupted sutures [43]. Both kidneys of recipient rats were nephrectomized at the time of RTx.

### Measurement of blood samples and survival

Blood samples were obtained for assessment of BUN and Cre on day 0 and each other day after RTx (AU400, Beckman Coulter, Inc., Brea, CA, USA). Each time, samples were centrifuged for 5 min at 3000 rpm, after which serum was collected. Survival of recipient rats was checked daily, until a maximum of 14 days, at which point rats were sacrificed.

### Evaluation of histopathological status

Following euthanasia at 14 days post-RTx, only the kidneys of rats surviving until day 14 were harvested and fixed in 10% formalin, and embedded with paraffin. Two-micron paraffin-embedded sections were stained with azan stain for assessment of parenchymal fibrosis. In accordance with previous studies [44], [45], fibrosis was graded numerically as none (0), minimal (1), moderate (2), and severe (3). The score index of fibrosis is shown in Figure 5. All slides were evaluated by four blinded observers, each of whom evaluated 20 fields for each slide (4×20 fields).

### Gravimetric measurements of kidney weights

Heparinized rats were euthanized by cervical dislocation, and their kidneys were left inside the body for 1 h. Left and right kidneys were perfused with the respective preservation solution (ETK or UW) at 4°C via the renal artery and kidney weights were measured (control). Subsequently, these kidneys were preserved for 2 h with the same solution at various temperatures (4, 23, 37°C). Kidney weights were measured by precision balance after removal of excess solution. These data were expressed as a percentage of control.

### Analysis of energy charge in preserved rat kidney

Fresh kidneys (fresh, n = 4 per group) or kidneys left for 1 h in the body after CD (CD1h, n = 4 per group) were nephrectomized and then preserved with ETK or UW at various temperatures (4, 23, and 37°C). One gram of kidney tissue was added to the chilled 0.5 N perchloric acid (5 mL), and then homogenated and centrifuged. This supernatant was added to 0.5 N triethenolamine 2.0 M K_2_CO_3_ before being separated by centrifugation. The control condition included no preservation of the kidney. For measurements of ATP, ADP, and AMP, aliquots of 50 µL were applied to an HPLC system using a 15-cm Inertsil ODS-3 column (GL Science Inc., Tokyo, Japan) at UV260 nm with a mobile phase containing 10 mM KH_2_PO_4_ with methanol. The results are expressed as nanomoles per milligram of protein. Total adenine nucleotide (TAN) and energy charge (EC) was calculated based on the following formulas:
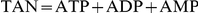


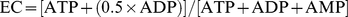



### Determination of fluorescence intensity in organ chips

Luc-Tg rats (n = 7 per group) were anesthetized with diethyl ether. One milliliter of 20% heparin saline solution was administered via the vein of the bulb of the penis or the inferior vena cava. After perfusion, the rats were exsanguinated, the kidney was removed and blood was rinsed with cold PBS. A kidney chip was made from each kidney and homogeneous organ samples with a diameter of 3 mm were prepared using a tissue slicer.

The organ samples were preserved in 96-well plates at a temperature of 4°C. Each plate was prefilled with 220 µL of the preservation solutions, including ETK, UW, and normal saline solution (Otsuka normal saline, Otsuka Pharmaceutical Factory, Inc.)

Fluorescence intensity of kidney samples after preservation was determined at specified time points using a plate reader (Mithras LB940, Berthold Japan, Tokyo, Japan) as previously described [46]. The plate reader was placed in the cold room, and the fluorescence intensity was measured at 4°C. A total of 20 µL of D-luciferin (2.29 mg/mL) was added to each well using an injector provided with the plate reader. The final concentration of D-luciferin in the well was 0.19 mg/mL, while the detection time was 1 s. The measurement was automatically repeated until the peak was detected, which was regarded as the fluorescence intensity. After the measurement, preservation solutions were immediately replaced.

### Detection and quantification of transgene expression using a non-invasive *in vivo* imaging system

*In vivo* luciferase imaging was performed using the noninvasive bioimaging system IVIS (Xenogen, Alameda, CA, USA) and IVIS Living Image (Xenogen) software package. The fresh or critical kidneys of Luc-Tg rats were transplanted into wild LEW rats respectively using a previously described rat RTx model. To detect photons from the Luc-Tg rat kidney, D-luciferin (Promega, Madison, WI, USA) was injected into the tail vein of the anesthetized recipient rat (30 mg/kg/body weight, dissolved and diluted to 15 mg/mL in PBS). The signal intensity was quantified as photon flux in units of photons/s/cm^2^ of a steradian in the region of interest [42], [46].

### Statistical Analysis

Data are presented as the mean ± SD. Survival curves were determined using the Kaplan-Meier method, while survival statistics were conducted by a Log-Rank test. Repeated measurements of BUN and Cre levels were compared between two groups using a Student's t-test, and among three or six groups using a nonparametric test (Kruskal-Wallis test). TAN, EC, and within-group variance of kidney weights were evaluated by Bartlett's test for equality of variance followed by Dunnett's test. Comparison between groups of different temperatures was performed by an F-test for homogeneity of variance followed by Student's t-test. The fluorescence intensity of kidney samples was tested by one-way analysis of variance (ANOVA) followed by Tukey post-hoc tests. Histopathological scores were evaluated by one-way ANOVA. A *P* value<0.05 was considered to be statistically significant.
